# Metabolomic Profiling in Lung Cancer: A Systematic Review

**DOI:** 10.3390/metabo11090630

**Published:** 2021-09-17

**Authors:** Daniela Madama, Rosana Martins, Ana S. Pires, Maria F. Botelho, Marco G. Alves, Ana M. Abrantes, Carlos R. Cordeiro

**Affiliations:** 1 Clinical Academic Center of Coimbra (CACC), Department of Pulmonology, Faculty of Medicine, University Hospitals of Coimbra, University of Coimbra, 3004-504 Coimbra, Portugal; carlos.crobalo@gmail.com; 2 Coimbra Institute for Clinical and Biomedical Research (iCBR), Biophysics Institute of Faculty of Medicine of University of Coimbra, Area of Environmental Genetics and Oncobiology (CIMAGO), 3000-548 Coimbra, Portugal; rfmartins@ua.pt; 3 Clinical Academic Center of Coimbra (CACC), Center for Innovative Biomedicine and Biotechnology (CIBB), Coimbra Institute for Clinical and Biomedical Research (iCBR), Biophysics Institute of Faculty of Medicine of University of Coimbra, Area of Environmental Genetics and Oncobiology (CIMAGO), 3000-548 Coimbra, Portugal; pireslourenco@uc.pt (A.S.P.); mfbotelho@fmed.uc.pt (M.F.B.); mabrantes@fmed.uc.pt (A.M.A.); 4 Department of Anatomy, Unit for Multidisciplinary Research in Biomedicine (UMIB), Institute of Biomedical Sciences Abel Salazar (ICBAS), University of Porto, 4099-002 Porto, Portugal; alvesmarc@gmail.com

**Keywords:** lung cancer, metabolomics, immunotherapy

## Abstract

Lung cancer continues to be a significant burden worldwide and remains the leading cause of cancer-associated mortality. Two considerable challenges posed by this disease are the diagnosis of 61% of patients in advanced stages and the reduced five-year survival rate of around 4%. Noninvasively collected samples are gaining significant interest as new areas of knowledge are being sought and opened up. Metabolomics is one of these growing areas. In recent years, the use of metabolomics as a resource for the study of lung cancer has been growing. We conducted a systematic review of the literature from the past 10 years in order to identify some metabolites associated with lung cancer. More than 150 metabolites have been associated with lung cancer-altered metabolism. These were detected in different biological samples by different metabolomic analytical platforms. Some of the published results have been consistent, showing the presence/alteration of specific metabolites. However, there is a clear variability due to lack of a full clinical characterization of patients or standardized patients selection. In addition, few published studies have focused on the added value of the metabolomic profile as a means of predicting treatment response for lung cancer. This review reinforces the need for consistent and systematized studies, which will help make it possible to identify metabolic biomarkers and metabolic pathways responsible for the mechanisms that promote tumor progression, relapse and eventually resistance to therapy.

## 1. Introduction

Lung cancer is still a burden in modern societies [1], and remains the leading cause of death by cancer worldwide. With 2.2 million new cases in 2020, or 12% of all diagnosed cancers, it is the second most commonly diagnosed form of the disease. Despite a small decline in the Western world, lung cancer incidence and mortality statistics are still increasing. Although smoking is one of the main risk factors, accounting for 25% of cases, 15% of lung cancers in men and 53% in women are not smoking-related [2].

When the cancer is diagnosed at an early stage, the five-year survival of patients is about 50%. However, more than 61% of patients are diagnosed in later stages (III and IV), when therapeutic options are limited. In this setting, the five-year survival can be as low as 4%. These statistics provide a strong motivation for the search for biomarkers that could aid the early detection of lung cancer or the provision of personalized treatments which take into consideration the treatment regimens available. To date, no such biomarkers have been clinically utilized, though it is a hot topic of research.

For many years, lung cancer therapy has been based on classical chemotherapy (CT) regimens, as well as radiotherapy and surgical therapy, depending on the stage of the disease. More recently, tyrosine kinase inhibitors (TKI) have emerged, revolutionizing the therapeutic regimens. The relationship between cancer and the immune system has been the subject of recent studies, which have brought to light the molecular mechanisms used by cancer cells to incorporate certain T-cell receptors, preventing the cytotoxic response which enables the defense from the antitumor immune attack [3]. ICIs are among the most significant improvements of the last decade in cancer treatment. Results obtained with programmed cell death protein-1 (PD-1) and programmed death-ligand 1 (PD-L1) inhibitors have demonstrated remarkable and durable clinical activity in patients with advanced non-small cell lung cancer (NSCLC). Such impressive responses have led to changes in the ongoing therapeutic algorithm of advanced NSCLC, with a new first-line treatment option for patients with tumors positive for PD-L1 [4] and a second-line option after an initial CT regimen. In recent years, randomized trials using ICIs showed positive results compared with standard CT. Although responses are surprising and durable, they are observed in 20–25% of unselected patients, which highlights the need for predicting factors and biomarkers for efficacy [3].

As described in 2000 by Hanahan and Weinberg, tumor development involves a complex network of events, including oncogene activation, insensitivity to anticancer signaling and evasion of apoptosis, high replicative potential, sustained angiogenesis and metastasis, and metabolic dysregulation [5]. These authors called these characteristics the hallmarks of cancer. More recently, two additional hallmarks were established in the pathogenesis of some and perhaps all cancers. The altered metabolism of cancer cells, which has been recognized as an emergent hallmark of cancer, is due to changes in signaling pathways, protein expression and other molecular mechanisms, along with specific biochemical adaptations during the carcinogenic process, which involve extensive interconnected events and important feedback loops. This means that the metabolic status of tumor cells gives them survival advantages in the process of tumor development. The second new hallmark allows malignant cells to bypass immunological destruction by T- and B-lymphocytes, macrophages and natural killer cells [5].

Tumor cells are characterized by consuming more glucose than regular cells and by their higher glycolytic capability and lactate production rate, even in the presence of oxygen. It is known that malignant cells reprogram their energy-production mechanisms, by using glycolysis even in normoxic conditions, leading to a state of “aerobic glycolysis”. This phenomenon is referred to as the “Warburg effect”. Various metabolomic changes in cancer cells being well-documented, of particular interest is the upregulated glycolysis, glutaminolysis and amino acid and fatty acid synthesis pathways. These metabolic pathways take advantage of the available cellular and environmental material to obtain energy and produce biomass to support the large-scale biosynthesis necessary for increased growth and proliferation [6].

As has already been said, there are numerous pathways involved in carcinogenesis, in addition to glycolysis. Glutaminolysis, which catabolizes glutamine in order to generate ATP and lactate, is one of those important pathways. Metabolic precursors are used by tumor cells for the synthesis of cellular blocks, essential for the maintenance of their increased proliferative capacity. One emblematic example includes the glycolytic intermediates used in the pentose phosphate pathway (PPP), as well as the nonessential amino acids used for nucleotide biosynthesis and derived from glucose and glutamine catabolism [7].

Over the last few years, the interest of metabolomics for the study of lung cancer has increased. One of the advantages of this technique is the possibility of using different types of samples, such as cultured cells, tissues and biofluids (e.g., blood plasma and serum, urine, bronchial aspirate, pleural fluid and exhaled breath condensate). More than 150 metabolites have been related to lung cancer-altered metabolism. In these studies, several biological samples and different metabolomic analytical platforms were used. Some results have been consistent in several published studies, showing the presence/alteration of specific metabolites. However, there is a clear variability due to lack of a full clinical characterization of patients or the selection of standardized patients. In addition, few studies have focused on the effect of cancer therapy on the evolution of the metabolomic profile—information that would be useful in developing personalized treatments.

In this systematic literature review, the authors reviewed the available evidence from published observational studies on the association between metabolites identified through metabolomics studies and lung cancer risk.

## 2. Methods

### 2.1. Literature Search Strategy

This systematic literature review was based on the Preferred Reporting Items for Systematic Reviews and Meta-analyses (PRISMA) guidelines [8] (Appendix A). And a review protocol was entered into the Prospero database (registration number 274188). A search in PubMed database was conducted which covered the past 10 years, until January of 2021. The search terms used included “metabolomics”, “lung cancer” and its variants (Appendix B). The research strategy was constructed considering the “PICO” method (Population/Patients, Intervention, Comparison, Outcome). The studies included evaluated patients (P) with NSCLC, and were studied in order to identify metabolomic alterations (I). In most of the articles, the results were compared with a healthy population (C). The outcome (O) was then evaluated in order to identify possible biomarkers, or differences which could facilitate the NSCLC diagnosis and treatment.

Only articles written in English were considered. Titles and abstracts were screened before searched articles were considered as potentially relevant for further evaluation. The full text was then evaluated before making the decision to include the study. Additional articles that emerged during the systematic analysis of the publications initially considered were also included.

### 2.2. Study Selection Criteria

Observational studies were included in this review if they reported at least one altered metabolite; if they reported either the estimated hazard ratio (HR), the odds ratio (OR), the risk ratio (RR) or the area under curve (AUC) for the association between the levels of the metabolite and any type of lung cancer; and if they reported the difference between the concentration of the metabolite in lung cancer patients compared to the controls. Studies were excluded if they were non-clinical studies, if sufficient data for analysis were not provided, or if they were not published in English.

### 2.3. Data Extraction

For the eligible studies, data were selected in a systematic way and using a previously elaborated registration form. For each study, significant data were registered, such as study design, the characteristics of participants, the type of identified metabolites, the type and stage of lung cancer, the type of biological samples collected for the quantification/identification of the metabolite, the analytical techniques used and the number of participants. When the studies reported an association between levels of a given metabolite and lung cancer, the HR/OR/RR/AUC and 95% confidence interval (CI) were analyzed. In some studies, relevant data were presented in graphical form. In these cases, the data were calculated based on the graphical data presented in the paper. Data were then grouped according to the identified metabolite and the type of biological sample.

## 3. Results

### 3.1. Eligible Studies

**Figure 1:**
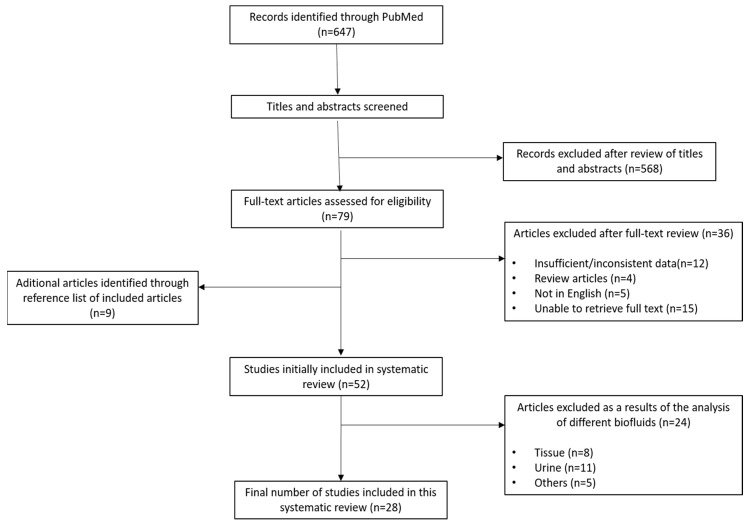
Flowchart of the literature search process and selection of studies.

The literature search process is presented in Figure 1. The literature search strategy allowed the identification of 647 studies, from which 79 were included for full-text review. During this process, 36 publications were excluded (for various reasons), and another 9 publications were added after consulting the references of the studies already included. In the final process, a total of 52 studies were included in the initial systematic literature review.

After the initial analysis of these 52 studies, it was concluded that a reduction of the search spectrum would be beneficial for the systematization of the results. Considering the volume of information obtained, it was decided to restrict this systematic review to studies that included only the results of the metabolomic profiles of the blood, plasma and serum of patients. As a result, 28 studies were classified as eligible.

The modified Newcastle-Ottawa Quality Assessment Scale for cross-sectional studies was used to assess the risk of bias for the included studies [9] (Table 1). The methodological quality score was calculated based on three domains: Selection, Comparability, and Exposure. To the assessment of each domain, a series of multiple-choice questions were answered after the reading of each study. A study could be classified with a maximum of one point for each numbered item within the Selection (four items), Exposure (three items) and Comparability (two items). Therefore, the scores could vary from a minimum of zero to a maximum of nine points.

### 3.2. Study Characteristics

Studies published between 2011 and 2021 were included (Table 2). A total of 28 studies were included in this systematic review. The majority of the studies included a healthy control group (*n* = 23). These studies were conducted in order to compare the metabolomic profiles of NSCLC patients and healthy patients. The five studies that did not include a healthy control aimed to evaluate the effect of therapy on the evolution of the metabolomic profile. However, in order to achieve this objective, these studies included two groups: a discovery group and a validation group (which was used as a control group).

Regarding the analytical method used to study the metabolomic profiles, eleven studies used liquid chromatography–mass spectrometry (LC–MS), two used gas chromatography–mass spectrometry (GC–MS), two used nuclear magnetic resonance spectrometry (NMRs), three electrospray ionization–mass spectrometry (ESI–MS), and one was an enzyme-linked immunosorbent assay (ELISA). Eight studies used more than one technique and one study did not specify the technique used.

In Table 3, the main results from each study are summarized. The majority of the enrolled studies included patients with a mean age over 60 years old (*n* = 16). In 21 of the studies included in this review, the population was mostly male. Regarding smoking habits, the majority of patients were current or former smokers. The studies were carried out in several countries, namely, China (*n* = 7), USA (*n* = 4), European countries (*n* = 9), Japan (*n* = 2), Canada (*n* = 2), Singapore (*n* = 1), and also in more than one country (*n* = 3). The metabolomic results of the enrolled studies are discussed in detail in the next section.

## 4. Discussion

Neoplastic disease is mainly characterized by a chronic and often uncontrolled cell proliferation, which depends on the deregulated control of cell proliferation and on the adjustments of energy metabolism needed to surpass the critical metabolic requirements for cell growth and division. In general, metabolism in these cells has many different particularities, for example, it is characterized by increased rates of glycolysis and/or glutaminolysis and biosynthetic processes that afford essential building blocks.

Metabolomics is a unique approach that attempts to decipher the interactions among transcriptional- and translational-level data, with a focus on the end result. Each type of cell and tissue has a characteristic metabolic composition that is uniquely altered in response to physiological and pathophysiological stimuli. These particular compositions reflect the collective effects of epigenetic factors, heterogeneous distributions of molecules and differential reaction rates. The metabolome of a sample—that is, the concentrations of the metabolites at a given time—can be thought of as a metabolic “fingerprint” representative of the state of the organism at a certain time. Metabolomics involves the quantification of metabolites, often in a temporal manner, to track the developing response to a stimulus [38].

The metabolome has the advantage of being much more dynamic than the proteome or genome, since metabolomics allows the detection of alterations in metabolites resulting from physiological and/or environmental events in shorter times [39]. Metabolomics integrates the effects of exogenous exposures (dietary intake, medication, lifestyle, variability in sample handling and preparation) [40], endogenous metabolism (e.g., co-morbidities such as diabetes and cardiovascular diseases) and genetic variations. Metabolomics is thus a powerful approach for detecting temporal physiological changes in real time and for monitoring potential environmental insults, disease progression and pathways that contribute to disease development, and for predicting the risk of disease or drug response. The level of detail that metabolomics provides could be particularly important in the efforts to obtain reliable and reproducible biomarkers [41,42,43].

As has already been mentioned, the interest in metabolomics for the study of cancer has grown over the years. The first studies to be developed included the analysis of tissue samples, while over the past several years more studies have been performed using biofluid analysis. The most studied biofluids are urine and blood serum/plasma. As far as the analytical techniques employed are concerned, most studies have used NMR, while biofluids have been examined using MS methods, due to their higher sensitivity. In the past decade, a considerable number of metabolomic studies have been performed that have aimed at making a tentative approach to identify potent and reliable biomarkers for lung cancer diagnosis. These studies used mainly plasma, serum, or urine. This systematic literature review aimed to provide a summary of the ongoing understanding of the association between metabolites and lung cancer risk, as well as the possibility of using new emergent biomarkers in lung cancer diagnosis.

### 4.1. Amino Acids

Several studies have been published focusing on the variation of amino acids in the metabolism of lung cancer cells. Kami et al. published that tumor concentrations of malate, fumarate and succinate were significantly higher in lung cancer patients than in normal individuals [44]. Xie et al. tried to identify probable biomarkers to diagnose lung cancer earlier. A combination of six metabolites was identified in this work. These metabolites were note-worthy for enabling the discrimination between stage I lung cancer patients and healthy individuals. The metabolites identified were proline, l-kynurenine, spermidine, amino-hippuric acid, palmitoyll-carnitine and taurine, with significant values of specificity and sensitivity [10]. Ni et al. conducted a similar study and identified glycine, valine, methionine, citrulline and arginine as amino acids with a strong ability to identify lung cancer [22,23]. Mazzone et al. also published results showing differences in 22 amino acids between healthy controls and lung cancer patients. Ten amino acids had higher values in lung patients (e.g., aspartate, N-acetylalanine, N-acetylmethionine…) and 12 had lower values (e.g., sarcosine, alanine, histidine, tyrosine, tryptophan…) [11]. Shingyogi et al. [19] and Miyagi et al. [17], also identified plasma free amino acid profiles that could be important in identifying lung cancer, including cancers at an early stage.

Data published by Puchades-Carrasco et al. showed that NSCLC patients exhibit higher serum levels of leucine/isoleucine, N-acetyl-cysteine and glutamate (37.65%) in comparison with healthy control individuals, in addition to lower levels of glutamine, threonine and histidine when compared with healthy individuals. These alterations were described in the serum metabolomic profiles of NSCLC patients at early as well as at advanced stages of the disease. The specific decrease of serum threonine and histidine levels observed in NSCLC patients can be attributed to the upregulation of the glycine/serine/threonine and pyrimidine metabolic pathways. Therefore, and corroborating previously published data, these molecular alterations are considered as metabolic hallmarks of NSCLC tumor-initiating cells as stated before. Furthermore, disease progression was found to be associated by these authors with a specific increase in the serum concentrations of lysine (increase of 13.16%), valine (21.05%) and phenylalanine (52.10%) [14].

#### 4.1.1. Carnitine and Cadaverine

Zhang et al. [16], reported in NSCLC patients increased plasma carnitine levels in all tumor stages. The increased production of this metabolite is described as a significant marker present in plasma, tumor tissue and in other types of biofluids collected from NSCLC patients. Furthermore, this metabolite has been already identified in patients with other types of cancer, such as bladder, breast and colorectal cancer [15]. Carnitine is rapidly utilized by tumor cells for acetyl-CoA synthesis and can be also used for lipid synthesis via glutamine metabolism. Zhang et al. reported another product of lysine metabolism, cadaverine, that is significantly increased in stage IIIB and IV NSCLC plasma patients. Influencing lysine metabolism, the abnormal carnitine and cadaverine increase confirms the hypothesis that lysine metabolism dysregulation is a common feature of NSCLC, especially in the advanced stages [16].

#### 4.1.2. Methionine

The ongoing studies characterizing the role of methionine in cancer cell proliferation are important. It is well known this metabolite is involved in various important activities in cancer cells, such as nucleotide biosynthesis via the one-carbon metabolism pathway and protein synthesis. Relating specifically to lung cancer, several published studies suggested that plasma methionine concentration is decreased [45].

#### 4.1.3. Tryptophan

As with methionine, the plasma levels of tryptophan have been described as low in lung cancer patients. In the kynurenine pathway, Tryptophan is a precursor molecule which synthesizes several metabolites that have immunosuppressive properties. The supression of T-cell proliferation and the transformation of natural killer cells were identified functions. Work carried out by Heng et al. [46] discovered that increased expression of indoleamine-2,3-dioxygenase 1 (IDO1) is positively correlated with worse lung cancer prognosis. This enzyme is involved in the synthesis of kynurenine from tryptophan, tryptophan and the kynurenine/tryptophan ratio being associated with a higher lung cancer risk, particularly after adjusting these values for established risk factors [13].

#### 4.1.4. Proline

This metabolite, a source for cellular energy production and an intermediate between the urea cycle and Krebs cycle, was identified in lung cancer patients’ plasma. Although the biochemical context of proline increase is unclear, the role of proline in lung carcinogenesis is an area of active research [47]. Several studies have identified that overexpression of proline dehydrogenase promotes cancer progression, however, more research is needed to characterize the role of this metabolite in cancer promotion and progression [48,49,50].

#### 4.1.5. Glutamine

Berker et al. compared serum profiles of prolonged and short survival groups of NSCLC patients. The data obtained showed that the prolonged survival cases demonstrated increased levels of glutamine, valine and glycine, and lower expressions of glutamate and lipid droplets. Glutamine is an essential metabolite for anabolic metabolism in cancer cells, and a higher consumption of glutamine has been described in cancer cells.

Tumor cells use the enzyme glutaminase to convert glutamine to glutamate and to produce precursors for the glutathione synthesis pathway, as well as using it in fatty acid production, which stimulates tumorigenesis. Regarding this metabolite, it is important to note that glutamine is an important energy source for cancer cells, particularly when glucose availability is scarce. Cancer cells use this metabolite as a supply for tumor growth, particularly in the short survival cases characterized by a rapid-growing lung cancer, which can explain the decreased levels of glutamine in short survival individuals [51,52].

The finding of higher glutamine levels in prolonged survival patients coheres with our knowledge of carcinogenesis and the biology of cancer. When analyzed, the blood of healthy individuals is found to contain high levels of glutamine as a ready source of carbon and nitrogen to sustain biosynthesis, energetics, and cellular homeostasis [52].

#### 4.1.6. Valine and Glycine

Several studies showed that NSCLC is associated with a significant increase in valine uptake. The increased blood valine concentration observed in the prolonged survival cases can be explained by the lower uptake of valine when compared with short lung cancer survival cases. The same hypothesis can be shared with glycine metabolites, respecting the increase of serum glycine levels when comparing the prolonged with the short survival cases. Glycine provides the carbon units to fuel the one-carbon metabolism for the synthesis of nucleic acids, proteins or lipids. Similar data reporting the association between higher levels of glycine and poorer prognoses were also reported for other types of cancer, such as human breast cancer, by Mahong Wu et al. [52].

### 4.2. Proteins

Li et al. developed a 13-protein lung cancer classifier from a panel of 371 protein candidates, previously identified in 143 plasma samples obtained from patients with benign and malignant lung nodules. This 13-protein classifier included proteins such as COIA, BGH3, LRP1, TSP1 and TETN, which were identified on an independent set of plasma samples (*n* = 104), with 90% negative predictive value and a specificity value of 44 ± 13% [12].

### 4.3. Lipids

Lipids, especially phospholipids and sphingolipids, play an essential structural and regulatory role in the formation of cellular membranes. Of particular interest, lipids also act as mediators in intra- and intercellular communications. Therefore, the lipidome is a crucial source of biomarkers for identifying a number of human diseases, including cancer [17].

Lower plasma lipid metabolites have been associated with lung cancer progression, since they influence tumor cell proliferation, progression and even dissemination. In a study by Terlizzi et al., reduced lipid contents were found in NSCLC patients’ blood. Higher levels of lipid contents were also observed in NSCLC patients caspase-4-positive [18]. Both circulating and tissue-associated caspase-4 were described by the authors as a diagnostic tool for NSCLC patients, since these patients had elevated levels of circulating caspase-4 [53]. It was found that 82.7% of plasma caspase-4 positive NSCLC patients had elevated levels of plasma lactate dehydrogenase (LDH). LDH is an enzyme recognized as a marker associated with tumor progression and prognosis, and which is also associated with tumor cell necroptosis or the anaerobic pattern established in the tumor mass. Circulating caspase-4 was, according to some authors, correlated with an increase in LDH. However, this effect was not described for all caspase-4 positive tumor tissues. In some cases, fatty acid biosynthesis was favored via malonic acid and palmitic acid production. It has been concluded by Terlizzi et al. that the caspase-4 positive subpopulation of NSCLC patients is characterized by a specific lipidomic profile associated with alternative pathways, which guarantee glucose metabolism in favor of tumor cell proliferation [18].

Mazzone et al. also published results showing differences in 68 lipids, between healthy controls and lung cancer patients. Forty-four different lipids had higher values in lung patients and 24 had lower values [11]. Yu et al. described significant discriminatory lipid species upregulated in lung cancer patients, such as lysophosphatidylethanolamine and ether phosphatidylethanolamine, as well as some downregulated lipids, such as sphingomyelins [20]. Another study by Ros-Mazurczy et al. identified phospholipid components at increased levels in lung cancer patients, including phosphatidylcholines, diacylophospholipids and sphingomyelins, as well as decreased levels of lysophosphatidylcholines [32].

Another study conducted by Klupczynska et al. presented choline-containing phospholipids as a promising group of lung cancer biomarkers, namely, lysophosphatidylcholine aC26:0, lysophosphatidylcholine aC26:1, phosphatidylcholine aaC42:4 and phosphatidylcholine aaC34:4 [26].

### 4.4. Glucose and Its Metabolites

In a study developed by Wikoff et al., significant elevations in ribitol, arabitol, and fucose/rhamnose in adenocarcinoma (AdC) tissues were observed, while a decrease in glucose levels was observed in tumoral tissues compared to non-malignant tissues [54]. The observed two-fold reduction in glucose in AdC when compared with healthy controls corroborates the Warburg effect theory. The study found that while glucose was reduced, other members of the glucuronate and pentose interconversion pathway (arabitol, ribitol, UDP-GlcNAc and xylitol) showed significant elevations in AdC compared to non-malignant tissues, suggesting that in cancer patients there is a pentose phosphate metabolism and an elevated glucuronidation activity. The pentose phosphate pathway is involved in nucleotide synthesis for DNA replication as well as the provision of reducing equivalents for several cellular reactions [55].

### 4.5. Smoking-Related Metabolites: Nicotine and Cotinine

Smoking is one of the most significant risk factors for lung cancer. From over 5000 compounds identified in tobacco smoke, more than 70 compounds have been considered carcinogenic.

Cotinine is one of the most important metabolites from nicotine metabolism in humans, being responsible for 70–80% of the metabolites produced in smokers [56,57]. Although the association between nicotine and cotinine levels and lung cancer risk is known, they are not considered carcinogenic. Several studies have shown that nicotine and cotinine did not induce or influence lung carcinogenesis. However, some data published by Larose et al. [58] on current smokers showed a significant positive association between cotinine and lung cancer risk. Cotinine levels compatible with active smoking were found to be usual in former smokers and never-smokers. Former and never-smokers with cotinine levels indicative of active smoking also displayed an increased risk of lung cancer. The authors concluded that there is a consistent association between circulating cotinine concentrations and lung cancer risk for current smokers and an additional risk in former and never-smokers [59,60,61,62,63].

### 4.6. N–Acetylneuraminic Acid (NANA)

Several studies associated the increased of NANA concentrations with various cancer types, including with higher lung cancer risk. NANA is involved in the formation of glycans, which plays several important roles in cells, namely in proteins synthesis, cell signaling and adhesion. Increased NANA levels may be a result of increased turnover and shedding of tumor cells, which in turn results in the release of glycans into the serum [45].

### 4.7. Folate and Vitamin B6

Fanidi et al. developed a case-control study with 5364 lung cancer patients and 5364 control subjects. Control participants were individually matched to lung cancer patients by age, sex, cohort and smoking status. This study concluded that participants with higher circulating concentrations of folate and vitamin B6 had a modest decreased overall risk of lung cancer [31].

### 4.8. Published Results Including Groups/Panels of Discriminative Metabolites

Sun et al. carried out a study including 31 lung cancer patients and 29 healthy volunteers, from which blood serum samples were collected. Levels of serum metabolites were qualitatively described with GC–MS. The authors identified five significant metabolites (erythritol, indole-3-lactate, adenosine-5-phosphate, paracetamol, and threitol) that could be further studied as biomarkers. In addition, they also identified five significant pathways, namely, pathways involving the metabolism of starch and sucrose, galactose, purine, tryptophan, and fructose and mannose degradation. These pathways can be altered and should be the subject of future in-depth studies, since they could serve to guide new investigations [27].

Singhal et al. developed an analysis of some metabolites that could be used to distinguish between lung cancer patients and healthy controls. The results reported by these authors described unsurprising changes in the concentrations of certain identified metabolites. For instance, lysophosphatidylcholines are membrane lipids, levels of which can reveal pathophysiological changes and which can be upregulated in lung cancer patients. Moreover, higher concentrations of several amino acids, such as valine, leucine and isoleucine, are also usually detected during the development of lung cancer. Increased levels of these amino acids are required for energy production through the Krebs cycle. It was also found that the metabolite diacetylspermine, used in this study to discriminate patients from controls, seems to be an excellent predictor of the diagnosis of NSCLC [29].

Mazzone et al. also showed differences between healthy controls and lung cancer patients in 12 peptides, 4 carbohydrates, 5 nucleotides and 30 xenobiotics [11]. Some of these metabolites presented higher values in lung cancer patients, while others presented lower values.

Cancer-associated biochemical alterations were characterized in the study conducted by Wikoff et al. In this study, current or former smokers with early stage (Stage IA–IB) AdC were included, and 39 malignant and non-malignant lung tissue samples were analysed by GC–MS. After analysis of 462 different metabolites, the main findings included decreased glucose levels; changes in cellular redox status associated with higher cysteine levels and levels of the antioxidants alpha- and gammatocopherol; increases in nucleotide metabolites 5,6-dihydrouracil and xanthine, which may be related to increased dihydropyrimidine dehydrogenase and xanthine oxidoreductase activity; higher 5′-deoxy-5′-methylthioadenosine levels, suggestive of reduced purine salvage and increased de novo purine synthesis; and elevations in glutamate and UDP-N-acetylglucosamine, suggesting increased protein glycosylation [54].

Comparisons of results from lung cancer cases against control groups (using serum, urine and bronchoalveolar lavage) revealed changes in 26 metabolites in serum, 32 in urine and 16 in the bronchoalveolar lavage. Six metabolites were commonly altered in the three fluids: malonic acid, palmitic acid, phosphoric acid, inositol, isocitric acid and l-glycine. From these, only phosphoric acid presented good sensitivity and specificity for lung cancer detection in the three fluids. In addition, this study showed alterations in different metabolic pathways, namely, glycine, serine and threonine metabolism; arginine and proline metabolism; inositol phosphate metabolism; alanine, aspartate and glutamate metabolism; pyruvate metabolism; galactose metabolism; and cysteine and methionine metabolism [64]. These are the main altered pathways in all the studies that have been referred to previously.

Results published by Rocha et al. showed that major characteristic alterations found in AdC patients were related to phospholipid metabolism (proved by findings of elevated levels of phosphatidylcholine, glycerophosphocholine and phosphatidylethanolamine) and protein catabolism. On the other hand, Squamous Cell Carcinoma (SqCC) patients had stronger glycolytic and glutaminolytic profiles (as shown by negative correlated variations in glucose and lactate levels and positive correlated increases in glutamate and alanine levels). This study provided new and clear evidence on the distinct metabolic signatures for AdC and SqCC, which can in turn have important implications for the differential diagnosis and selective definition of new targeted therapeutics [65].

### 4.9. Metabolites and the Response to Treatment

There have been many publications presenting studies of the role of metabolomics in lung cancer diagnosis, tumor characterization and progression. However, there is little information about the relation between serum metabolomic profiles and the clinical outcomes of advanced-stage lung cancer patients. To fill the gap, several studies have been conducted, such as the one carried out by Maosheng et al., which intended to examine the role of blood metabolites in predicting overall survival among advanced-stage NSCLC patients who received platinum-based CT [33]. The authors found altered levels in four metabolites, namely, caffeine, paraxanthine, stachydrine, and methyl glucopyranoside-alpha and -beta, which levels differed significantly between NSCLC patients with poor and good chances of survival. Interestingly, most of these metabolites are involved in caffeine metabolism, two of these metabolites related to coffee intake. Although the mechanisms remain unclear, the caffeine metabolism pathway was the only significant pathway identified, exhibiting differences between NSCLC patients with poor and good survival chances, after receiving platinum-based CT. These small metabolites may be useful biomarkers in the process of identifying patients who may benefit from platinum-based CT [33].

Animal experimental models reported an antineoplastic effect of caffeine, inducing cell differentiation and inhibiting mitosis in tissue cultures. Three significant metabolites involved in the caffeine metabolism pathway and identified by Guertin et al. have been inversely associated with colorectal cancer, namely theophylline, caffeine and paraxanthine [66]. Moreover, caffeine and/or coffee intake has been negatively associated with breast tumor differentiation, suggesting that they may slow tumor growth [67]. However, there are very few published results about caffeine and its metabolites and lung cancer.

In addition, caffeine exposure may sensitize tumor cells to ionizing radiation [68]. Some authors published results showing that metabolites of the caffeine metabolism pathway may deregulate cell cycle checkpoints and phosphorylation of p53 in NSCLC patients who received CT and radiotherapy, influencing their survival. The underlying mechanisms and the association between caffeine and its metabolites and the survival of NSCLC patients treated with CT requires further study.

Tian et al. [34] recently reported interesting findings from metabolomic profiles from a cohort of NSCLC patients treated with platinum-doublet CT. This study included pre-treatment serum metabolomics profiling after first-line pemetrexed plus platinum doublet, in order to explore a potential biomarker model predictive of treatment efficacy and survival outcomes. The metabolite panel reported by Tian et al. included seven metabolites (hypotaurine, uridine, dodecanoylcarnitine, choline, dimethylglycine, niacinamide, L-palmitoylcarnitine). The presence of these metabolites in the pre-treatment serum was associated with longer median progression free survival (10.3 vs. 4.5 months, *p* < 0.001).

In another work conducted by Hao et al., it was postulated that increased glutathione synthesis resulting from upregulated methylation pathways could be associated with better survival of CT-treated NSCLC patients [35,69]. The authors positively associated increased blood levels of 2-hydroxybutyrate, glycine and formate with better overall survival. Sphingolipids were also positively associated with overall survival, namely sphingomyelin and two ceramides. Considering the presented results, the authors hypothesized that elevated ceramide synthesis from membrane sphingomyelin may be indicative of better overall survival and less aggressive tumorigenesis. The evaluation of metabolomic profiles from pre-treatment samples, may be a promising approach to stratifying clinical outcomes for NSCLC patients who will be receiving CT.

Another work published by Hao et al. evaluated the pre-treatment serum metabolomic profiles of 25 lung cancer patients undergoing CT ± radiation to understand the usefulness of metabolites as temporal biomarkers of clinical outcomes [36]. Hydroxylamine, tridecan-1-ol and octadecan-1-ol metabolites were suggestive of better survival, while metabolites such as tagatose, hydroxylamine, glucopyranose and threonine were indicative of tumor progression. These authors concluded that baseline samples, naive to treatment, are potentially predictive of essential clinical parameters, like survival and progression, and may reflect tumor pathophysiology (SqCC vs. AdC) and tumor stage.

More recently, Ghini et al. [37], used metabolomics to analyze sera samples from 50 patients with NSCLC treated with ICPIs. Significantly, this study showed that the metabolomic profiling of serum could be used as a predictive “collective” biomarker to ICPI response. Metabolomic profiling predicted, with >80% accuracy, the individual therapy outcome. Significantly different levels of the amino acids alanine and pyruvate were observed on nivolumab-treated patients, non-responder and responder subjects (*p* < 0.05). Responders revealed lower serum levels of the two metabolites mentioned. Considering pembrolizumab-treated patients, there was a tendency for pyruvate serum levels to be lower in non-responders compared with responders.

### 4.10. Limitations of this Study

Various lung cancer metabolic markers have been studied, but the published studies included only a limited number of clinical cases. Large-scale clinical validations, as well as in vitro targeted studies are absent, so the role of metabolomics in lung cancer diagnosis requires further study and validation. Most of the metabolites were screened by the comparison of values in lung cancer patients to those in healthy subjects, and the differentiation of lung cancer and other diseases lacks validation [70].

This review summarizes the most recent data on the metabolomic profiles for serum, blood or plasma of lung cancer patients. Despite the variety of metabolites associated with changes in lung cancer, the limited quantity of evidence found for each metabolite suggests that further studies are needed in order to validate the option of using them as biomarkers for lung cancer. Moreover, most studies focused on a qualitative comparison, and therefore quantitative analysis is lacking. Measure and report of absolute concentrations of the metabolic markers in tissues, cells, blood and urine is also missing. These aspects largely limit the clinical application of these results. As previously stated, there are few results regarding the effect of the different therapeutic approaches (chemotherapy, immunotherapy, combined therapies, radiotherapy) in metabolomic profiles, which leaves a gap in the evaluation of the importance and influence of metabolomics in lung cancer research.

Heterogeneity was noted in the identified studies. It may be attributed to endogenous and exogenous factors such as ethnicity, co-morbidities and smoking status, as well as dietary intake, medication, sampling collection and preparation. The results of a metabolomic evaluation are profoundly affected by these factors, so they act as a bias across all the articles included in this review. Moreover, the analytical platforms and methodology used to identify and quantify the metabolites should be further harmonized in order to standardize the workflow adopted by researchers in metabolomics research.

As an interdisciplinary subject, metabolomics must be integrated with many related areas, including analytical chemistry, molecular biology, biochemistry, bioinformatics and computer big data science. Specifically for lung cancer, software tools, big data integration and collection platforms that are able to integrate genomic, proteomic, metabolomic and imagiologic data are still not available.

## 5. Conclusions

Metabolomics is an emerging field compared with other, older technological methodologies, like genomics, transcriptomics and proteomics. Moreover, it has only been newly applied to the study of lung cancer. Although there are several difficulties related to the use of metabolomics in lung cancer research, some lung cancer metabolomic markers have been identified. Its identification in a limited number of clinical cases urges the need for large-scale clinical validations. Most of the metabolites identified as possible lung cancer biomarkers were selected on the basis of comparisons of metabolites in lung cancer patients and in healthy people, however, the differentiation of lung cancer and other diseases lacks validation. Moreover, for most metabolic markers, quantitative analysis is lacking, as is the testing of absolute concentrations of the metabolic markers. These factors largely limit the clinical application of metabolomics in lung cancer research.

Despite all the limitations in already published studies, this review identified several metabolites that are significantly associated with metabolic pathways responsible for mechanisms that promote tumor progression, particularly in lung cancer. Certain amino acids, proteins, smoking-related metabolites, folate and lipids are particularly promising, and should be considered in further investigations for potential use as biomarkers in larger populations, as well as in the study of relapse and resistance to therapy. It is also important to highlight that larger and standardized studies (in vitro and in vivo) using some of the metabolites already studied are needed in order to evaluate the influence of numerous factors such as diet and lifestyle patterns, systemic responses of the host (e.g., inflammation and response of the immune system) and the influence of and response to the different available therapies.

The authors predict and hope that in the future, metabolomics will play a more important role in the early diagnosis of lung cancer, in distinguishing between different types of tumors, in molecular mechanism research and in precision/targeted medicine, since it is an expanding branch of science and one that allows resort to the use of non-invasive and easy-to-collect samples.

## Figures and Tables

**Table 1:** Risk of bias for the included studies, using the modified Newcastle-Ottawa Quality Assessment Scale adapted for cross-sectional studies [9].

Authors, Year	Selection (0–4)	Comparability (0–2)	Exposure (0–3)	Risk of Bias (0–9)
Xie et al., 2021 [10]	4	2	2	8
Mazzone et al., 2015 [11]	4	2	2	8
Li et al., 2013 [12]	4	2	2	8
Chuang et al., 2014 [13]	3	2	2	7
Puchades et al., 2016 [14]	4	2	2	8
Klupczynska et al., 2017 [15]	3	2	2	7
Zhang et al., 2020 [16]	4	2	2	8
Miyagi et al., 2011 [17]	3	2	3	8
Terlizzi et al., 2018 [18]	3	2	2	7
Shingyogi et al., 2013 [19]	3	0	2	5
Yu et al., 2017 [20]	4	2	2	8
Skaaby et al., 2014 [21]	3	2	3	8
Ni et al., 2016 [22]	3	2	2	7
Ni et al., 2019 [23]	3	2	2	7
Larose et al., 2018 [24]	2	2	2	6
Pietzke et al., 2019 [25]	2	0	2	4
Klupczynska et al., 2019 [26]	2	2	2	6
Sun et al., 2018 [27]	4	2	1	7
Faharmann et al., 2015 [28]	4	2	2	8
Singhal et al., 2019 [29]	3	2	1	6
Wang et al., 2015 [30]	3	2	2	7
Fanidi et al., 2018 [31]	3	2	2	7
Ros-Mazurczyk et al., 2017 [32]	4	2	1	7
Maosheng et al., 2017 [33]	3	2	2	7
Tian et al., 2018 [34]	3	2	2	7
Hao et al., 2020 [35]	2	1	2	5
Hao et al., 2016 [36]	3	1	2	6
Ghini et al., 2020 [37]	3	2	2	7

**Table 2:** Summary of the characteristics of the studies included in the review.

Subject Groups (No. of Samples)		Analytical Technique	Objective of the Metabolomic Profile Analysis				Reference
Healthy Controls	NSCLC Patients		Compare Cancer vs. Control	Distinguish Histological Types	Disease Staging	Other	
**Blood, Serum and/or Plasma**							
43	110	LC-MS			×	Biomarker	Xie et al., 2021 [10]
190	94	LC-MS, GC-MS	×		×		Mazzone et al., 2015 [11]
71	72	NS	×			Early diagnosis	Li et al., 2013 [12]
893	1748	LC-MS, GC-MS	×			Biomarker	Chuang et al., 2014 [13]
114	182	NMRs	×		×		Puchades et al., 2016 [14]
25	50	LC-MS	×			Biomarker	Klupczynska et al., 2017 [15]
60	156	LC-MS	×		×		Zhang et al., 2020 [16]
200	996	ESI-MS	×			Early diagnosis	Miyagi et al., 2011 [17]
79	125	ELISA	×			Overall survival, Biomarker	Terlizzi et al., 2018 [18]
86	323	ESI-MS	×			Early diagnosis	Shingyogi et al., 2013 [19]
147	199	ESI-MS	×	×			Yu et al., 2017 [20]
10,485	126	UPLC-MS, Immunoassay	×				Skaaby et al., 2014 [21]
40	100	LC-MS	×				Ni et al., 2016 [22]
17	30	LC-MS	×				Ni et al., 2019 [23]
5364	5364	LC-MS	×			Biomarker	Larose et al., 2018 [24]
56	50	LC-MS, GC-MS	×				Pietzke et al., 2019 [25]
20	20	MS	×		×		Klupczynska et al., 2019 [26]
29	31	GC-MS	×				Sun et al., 2018 [27]
74	95	GC-MS	×				Faharmann et al., 2015 [28]
29	57	LC-MS	×			Treatment monitoring tool	Singhal et al., 2019 [29]
100	100	LC-MS	×		×		Wang et al., 2015 [30]
5364	5364	LC-MS, GC-MS	×			Risk factors	Fanidi et al., 2018 [31]
300	100	LC-MS	×				Ros-Mazurczyk et al., 2017 [32]
0	220	LC-MS				Overall survival, Treatment efficacy	Maosheng et al., 2017 [33]
0	354	LC-MS				Overall survival, Treatment efficacy	Tian et al., 2018 [34]
0	774	LC-MS, UPLC-MS, NMRs				Treatment efficacy	Hao et al., 2020 [35]
0	25	NMRs, GC-MS			×	Prognosis	Hao et al., 2016 [36]
0	50	NMRs				Treatment efficacy	Ghini et al., 2020 [37]

NSCLC: Non-Small Cell Lung cancer; CE–MS: Capillary electrophoresis—mass spectrometry; LC–MS: Liquid chromatography—mass spectrometry; GC–MS: Gas chromatography—mass spectrometry; NS: Non-specified; NMRs: Nuclear magnetic resonance spectrometry; ELISA: Enzyme-linked immunosorbent assay; ESI–MS: Electrospray ionization—mass spectrometry; MS: Mass spectrometry; UPLC–MS: Ultra-high performance liquid chromatography—mass spectrometry.

**Table 3:** Characteristics of the included studies, with the main alterations in metabolites/metabolomic profiles identified.

Subject Groups			Place Where the Study Was Carried Out	Identified Metabolites			Measure of Association	Effect Size	Reference
Age Mean	Gender Male (%)	Smoking Status		Amino Acids	Lipids	Others			
NS	NS	NS	China	Proline			AUC	0.989	Xie et al., 2021 [10]
				l-kynurenine spermidine amino-hippuric acid				Sensitivity = 98.1%	
				palmitoyll-carnitinetaurine				Specificity = 100.0%	
67	52%	S or FS 97%	USA	10 amino acids had higher values in lung patients and 12 had lower values	44 different lipids had higher values in lung patients and 24 had lower values	Differences in 12 peptides, 4 carbohydrates, 5 nucleotides and 30 xenobiotics between healthy controls and lung cancer patients			Mazzone et al., 2015 [11]
65	33%	S–14% N–6%	USA			13-protein lung cancer classifier			Negative predictive value (NPV) of 90%	Li et al., 2013 [12]
									specificity of 44 ± 13%	
59	62%	S–59% N–11%	Europe	Tryptofan Kynurenine				OR	0.88 (0.59–1.30)	Chuang et al., 2014 [13]
								OR	1.30 (0.92–1.84)	
63	87%	S–44% N–7%	Spain	Specific increase in the serum concentrations of lysine (13.16%), valine (21.05%) and phenylalanine (52.10%)				P	0.0025	Puchades et al., 2016 [14]
								P	0.0000	
								P	0.0000	
64 ± 6.9	64%	S–48% N–51%	Poland	Panel of 12 compounds, including some amino acids		Panel of 12 compounds, including acylcarnitine, organic acids		AUC	0.836 (0.722–0.946)	Klupczynska et al., 2017 [15]
42–79	38%	S–19% N–11%	China			β-hydroxybutyric acid, LysoPC 20:3, PC ae C40:6, citric acid, fumaric acid		AUC	>0.9	Zhang et al., 2020 [16]
65 ± 10	62.5%	S–42% N–30%	Japan	Profile of plasma free amino acids				AUC	0.75	Miyagi et al., 2011 [17]
60 ± 10	66%		Italy				Higher levels of Caspase 4 in NSCLC		Sensitivity: 97.07–100%	Terlizzi et al., 2018 [18]
									specificity 88.1%	
									positive predictive value of 92.54%	
									accuracy of 95.19%	
									AUC of 0.971	
67.8 ± 8.2	43%	S–34% N–21%	Japan	Profile of plasma free amino acids				AUC	0.731–0.806	Shingyogi et al., 2013 [19]
67 ± 8	54%	All S or FS	China and USA		Four lipid markers (LPE(18:1), ePE(40:4), C(18:2)CE and SM(22:0))			AUC	82.3%	Yu et al., 2017 [20]
18–71	48%	S–37% N–35%	Denmark				Vitamin D	HR	0.98 (0.91–1.05)	Skaaby et al., 2014 [21]
51–83	65%		China	Panel of 13 amino acids			Panel of 8 acylcarnitines			Ni et al., 2016 [22]
66.7	65%	S–23% N–47%	China	Glycine, valine, methionine, citrulline and arginine				p	0.033	Ni et al., 2019 [23]
									0.378	
									0.067	
									0.039	
									0.015	
60	54%	S–47% N–25%	Europe, USA, China				Cotinine	OR	S: 1.39 (1.32–1.47)	Larose et al., 2018 [24]
									FS: 1.17 (1.07–1.28)	
									N: 1.64 (1.10–2.30)	
66 ± 9	87.5%	S–50% N–50%	Europe				Formate levels higher in lung cancer patients			Pietzke et al., 2019 [25]
62 ± 5	55%	S–60%	Poland		Lysophosphatidylcholine aC26:0			AUC	0.87 (0.73–0.96)	Klupczynska et al., 2019 [26]
					Lysophosphatidylcholine aC26:1			AUC	0.84 (0.68–0.95)	
					Phosphatidylcholine aaC42:4			AUC	0.81 (0.65–0.93)	
					Phosphatidylcholine aaC34:4			AUC	0.82 (0.65–0.94)	
54.1 ± 9.9	67.7%	S–71%	China				Erythritol, indole-3-lactate, adenosine-5-phosphate, paracetamol, threitol	AUC	0.9	Sun et al., 2018 [27]
65.9 ± 9.7	62%		USA	Aspartate Glutamate					Sensitivity: 67.5%	Faharmann et al., 2015 [28]
									specificity 95.4%	
									Sensitivity: 70.9%	
									specificity 74.4%	
52	53%		USA and Canada	Valine			LysoPhosphatidylcholine acyl C18:2	AUC	0.97 (0.875–1.0)	Singhal et al., 2019 [29]
							decadienyl-L-carnitine			
							phosphatidylcholine			
							acyl-alkyl C36:0			
							phosphatidylcholine diacyl C30:2			
							spermine			
							iacetylspermine			
57.1 ± 8.6	52%	S–48%	China				25(OH)D deficiency → related to higher risk of NSCLC	P	0.03	Wang et al., 2015 [30]
		N–32%								
60	54%	S–47%	Singapore				Vitamin B6 and folate elevated → decreased risk	OR	0.88 (0.78–1)	Fanidi et al., 2018 [31]
		N–25%							0.86 (0.74–0.99)	
			Poland		Increased levels in lung cancer patients: phosphatidylcholines, diacylophospholipids and sphingomyelins; decreased levels of lysophosphatidylcholines			AUC	0.88	Ros-Mazurczyk et al., 2017 [32]
60.2	56.3%	S–44%	USA				Caffeine	P	<0.05	Maosheng et al., 2017 [33]
							paraxanthine			
							stachydrine			
		N–16%					methyl glucopyranoside (αβ)			
37–84	86%	N–55%	China				Hypotaurine	AUC	0.912	Tian et al., 2018 [34]
							uridine			
							dodecanoylcarnitine			
							choline			
							dimethylglycine			
							niacinamide			
		FS–45%					L-palmitoylcarnitine → longer PFS			
			Canada				Elevated blood 2-hydroxybutyrate, glycine, sphingomyelin and formate were positively associated with better OS			Hao et al., 2020 [35]
64	60%		Canada				Hydroxylamine, tridecan-1-ol	P	<0.05	Hao et al., 2016 [36]
							octadecan-1-ol → better survival Tagatose			
							hydroxylamine			
							glucopyranose			
	54%	S–34%	Italy	Alanine and pyruvate → responders were characterized by lower serum levels			threonine → progression	P	<0.05	Ghini et al., 2020 [37]
		N–5%								

SD—Standard deviation; S—current smoker; N—Never smoker; FS—former smoker; CI—Confidence Interval; OR—Odds ratio; AUC—Area under curve; HR—Hazard ratio; PFS—Progression free survival; OS—Overall survival.

## Appendix A

**Table A1:** PRISMA checklist, retrieved from PRISMA website. Some items were not included (X) since this is a systematic review and not a meta-analysis.

Section/Topic	#	Checklist Item	Reported on Section
**Title**			
Title	1	Identify the report as a systematic review, meta-analysis, or both.	Title
**Abstract**			
Structured summary	2	Provide a structured summary including, as applicable: background; objectives; data sources; study eligibility criteria, participants, and interventions; study appraisal and synthesis methods; results; limitations; conclusions and implications of key findings; systematic review registration number.	Abstract
**Introduction**			
Rationale	3	Describe the rationale for the review in the context of what is already known.	Introduction
Objectives	4	Provide an explicit statement of questions being addressed with reference to participants, interventions, comparisons, outcomes and study design (PICOS).	Introduction
**Methods**			
Protocol and registration	5	Indicate if a review protocol exists, if and where it can be accessed (e.g., Web address), and, if available, provide registration information including registration number.	X
Eligibility criteria	6	Specify study characteristics (e.g., PICOS, length of follow-up) and report characteristics (e.g., years considered, language, publication status) used as criteria for eligibility, giving rationale.	Section 2.2
Information sources	7	Describe all information sources (e.g., databases with dates of coverage, contact with study authors to identify additional studies) in the search and date last searched.	Section 2.1
Search	8	Present full electronic search strategy for at least one database, including any limits used, such that it could be repeated.	Appendix B Search 2
Study selection	9	State the process for selecting studies (i.e., screening, eligibility, included in systematic review, and, if applicable, included in the meta-analysis).	Section 2.2
Data collection process	10	Describe method of data extraction from reports (e.g., piloted forms, independently, in duplicate) and any processes for obtaining and confirming data from investigators.	Section 2.3
Data items	11	List and define all variables for which data were sought (e.g., PICOS, funding sources) and any assumptions and simplifications made.	Section 2.3
Risk of bias in individual studies	12	Describe methods used for assessing risk of bias of individual studies (including specification of whether this was done at the study or outcome level), and how this information is to be used in any data synthesis.	X
Summary measures	13	State the principal summary measures (e.g., risk ratio, difference in means).	Section 2.2
Synthesis of results	14	Describe the methods of handling data and combining results of studies, if done, including measures of consistency (e.g., I^2^) for each meta-analysis.	X

## Appendix B

Literature search strategy used for electronic database PubMed. The search performed was #1 AND #2, with the filter in the last 10 years → Search 3.

Search 1: lung cancer Filters: in the last 10 years

(“lung neoplasms”[MeSH Terms] OR (“lung”[All Fields] AND “neoplasms”[All Fields]) OR “lung neoplasms”[All Fields] OR (“lung”[All Fields] AND “cancer”[All Fields]) OR “lung cancer”[All Fields]) AND (y_10[Filter]).

Translations: lung cancer: “lung neoplasms”[MeSH Terms] OR (“lung”[All Fields] AND “neoplasms”[All Fields]) OR “lung neoplasms”[All Fields] OR (“lung”[All Fields] AND “cancer”[All Fields]) OR “lung cancer”[All Fields].

Search 2: metabolomics Filters: in the last 10 years

(“metabolome”[MeSH Terms] OR “metabolome”[All Fields] OR “metabolomes”[All Fields] OR “metabolomics”[MeSH Terms] OR “metabolomics”[All Fields] OR “metabolomic”[All Fields]) AND (y_10[Filter]).

Translations: metabolomics: “metabolome”[MeSH Terms] OR “metabolome”[All Fields] OR “metabolomes”[All Fields] OR “metabolomics”[MeSH Terms] OR “metabolomics”[All Fields] OR “metabolomic”[All Fields].

Search 3: (lung cancer AND (y_10[Filter])) AND (metabolomics AND (y_10[Filter])) Filters: in the last 10 years

(“lung neoplasms”[MeSH Terms] OR (“lung”[All Fields] AND “neoplasms”[All Fields]) OR “lung neoplasms”[All Fields] OR (“lung”[All Fields] AND “cancer”[All Fields]) OR “lung cancer”[All Fields]) AND “2011/03/03 00:00”:”3000/01/01 05:00”[Date–Publication] AND (“metabolome”[MeSH Terms] OR “metabolome”[All Fields] OR “metabolomes”[All Fields] OR “metabolomics”[MeSH Terms] OR “metabolomics”[All Fields] OR “metabolomic”[All Fields]) AND “2011/03/03 00:00”:”3000/01/01 05:00”[Date–Publication])) AND (y_10[Filter]).

Translations: lung cancer: “lung neoplasms”[MeSH Terms] OR (“lung”[All Fields] AND “neoplasms”[All Fields]) OR “lung neoplasms”[All Fields] OR (“lung”[All Fields] AND “cancer”[All Fields]) OR “lung cancer”[All Fields] y_10[Filter]: “last 10 years”[dp].

Metabolomics: “metabolome”[MeSH Terms] OR “metabolome”[All Fields] OR “metabolomes”[All Fields] OR “metabolomics”[MeSH Terms] OR “metabolomics”[All Fields] OR “metabolomic”[All Fields] y_10[Filter]: “last 10 years”[dp].
